# Effect of Different Flame-Retardant Bridged DOPO Derivatives on Properties of in Situ Produced Fiber-Forming Polyamide 6

**DOI:** 10.3390/polym12030657

**Published:** 2020-03-13

**Authors:** Jelena Vasiljević, Marija Čolović, Nataša Čelan Korošin, Matic Šobak, Žiga Štirn, Ivan Jerman

**Affiliations:** 1 Faculty of Natural Sciences and Engineering, University of Ljubljana, Aškerčeva 12, 1000 Ljubljana, Slovenia; 2 National Institute of Chemistry, Hajdrihova 19, 1000 Ljubljana, Slovenia; Matic.Sobak@ki.si (M.Š.); Ziga.Stirn@ki.si (Ž.Š.); ivan.jerman@ki.si (I.J.); 3 Faculty of Chemistry and Chemical Technology, University of Ljubljana, Večna pot 113, 1000 Ljubljana, Slovenia; natasa.celan@fkkt.uni-lj.si

**Keywords:** PA6, bridged DOPO derivatives, in situ polymerization, thermal stability, flame retardancy

## Abstract

The production of sustainable and effective flame retardant (FR) polyamide 6 (PA6) fibrous materials requires the establishment of a novel approach for the production of polyamide 6/FR nanodispersed systems. This research work explores the influence of three different flame-retardant bridged 9,10-dihydro-9-oxa-10-phosphaphenanthrene-10-oxide (DOPO) derivatives on the comprehensive properties of in situ produced PA6/FR systems. To this end, in situ water-catalyzed ring-opening polymerization of *ε*-caprolactam was conducted in the presence of three different bridged DOPO derivatives, e.g., one P−N bond phosphonamidate derivative and two P−C bond phosphinate derivatives. The selected bridged DOPO derivatives mainly act in the gas phase at the temperatures that relatively match the PA6 pyrolysis specifics. The effects of the FRs on the dispersion state, morphological, molecular, structural, melt-rheological, and thermal properties of the in situ synthesized PA6 were evaluated. The specific advantage of this approach is one-step production of PA6 with uniformly distributed nanodispersed FR, which was obtained in the case of all three applied FRs. However, the applied FRs differently interacted with monomer and polymer during the polymerization, which was reflected in the length of PA6 chains, crystalline structure, and melt-rheological properties. The applied FRs provided a comparable effect on the thermal stability of PA6 and stabilization of the PA6/FR systems above 450 °C in the oxygen-assisted pyrolysis. However, only with the specifically designed FR molecule were the comprehensive properties of the fiber-forming PA6 satisfied for the continuous conduction of the melt-spinning process.

## 1. Introduction

The semicrystalline linear polyamide 6 (PA6) polymer is one of the most important synthetic fiber-forming polymers because of its excellent processing properties and good mechanical and tribological properties. An additional advantage of the PA6 is its chemical recyclability, which enables the re-usability and the possibility for closing the loop on the circular economy [1]. The applications of PA6 textile filament yarns range from carpets, apparel, and upholstery to tire cords, seat belts, automotive and other transportation interior and seats covers, parachutes, tents and sleeping bags, etc. For such applications, increased thermal stability and flame retardancy are required in order to comply with fire safety regulations. 

To this aim, flame-retardant additives are commonly physically incorporated into the PA6 matrix during melt-processing in order to produce composite structures [2,3,4,5,6,7]. However, the strong inter- and intra-molecular attractive interactions between polymer chains inhibit obtaining the highly dispersed and uniformly distributed state of flame retardants in the PA6 matrix. Consequently, the effectiveness of the incorporated flame retardants is decreased, which imposes the inevitable use of high FR loading. Although high FR loading is commonly used in bulk plastics, in the melt-spinning process, it is undesirable as it impairs spinnability, causing clogging of filters and spinnerets, filament breakage, as well as significant reduction of the filament tensile properties. The complexity of this problem gains even higher magnitude when the limitations regarding the compatibility of the flame retardant with the processing parameters and PA6 pyrolysis specifics are considered. The flame retardant must be stable during polymer melt processing and its thermal decomposition should provide chemical interaction at temperatures that maximally match the polymer decomposition temperatures [8]. An additional limitation factor refers to the integration of the sustainability concept into the production and application of flame-retardant materials without compromising their performance. However, all of these limitations drive the development of new sustainable and effective flame-retardant alternatives for each specific polymer in order to prevent deaths and economic losses as well as impact on both the environment and society. 

The increasing concern and gradual banning of persistent, bioaccumulative, and toxic (PBT) flame retardants significantly contribute to the building of a non-toxic green environment [9]. Today, inorganic, phosphorus-, and nitrogen-containing flame retardants are considered as more sustainable alternatives to their PBT halogenated counterparts. Because of the chemical versatility and ability to effectively provide flame-retardant protection in both the condensed and gas phase, phosphorus flame retardants are continuously developed to improve the flame retardancy of polymers and meet sustainability standards [8,10]. As a promising non-halogenated alternatives, organophosphorus flame retardants have found application as physically incorporated low and high molecular weight additives [11,12,13,14,15,16,17] as well as being chemically bonded to the polymer [18,19,20]. Furthermore, the flame-retardant development based on using renewable bio-sources became particularly attractive [21,22,23]. Among the organophosphorus flame retardants, phosphinate, phosphonate, and phosphonamidate derivatives of 9,10-dihydro-9-oxa-10-phosphaphenanthrene-10-oxide (DOPO) derivatives have been intensively developed and investigated because of their versatile flame-retardant mode of action during a fire and application ranging from epoxy resins, polyurethane foams, engineering plastics, to polyester and polyamide fibers [24,25]. 

The recently proposed direction towards solving the flammability problem of PA6 textile materials is based on the production of copolyamides by using 3-hydroxyphenylphosphinylpropanoic acid and 9,10-dihydro-10-[2,3-di(hydroxycarbonylpropyl]-10-phosphaphenanthrene-10-oxide as comonomers [19]. Whilst the later comonomer enables the production of copolyamide PA6/PA6,4 with DOPO group as a side group, the former enables incorporation of the phosphorus element in the copolyamide back chain. Another example of copolyamide production was reported by using 2,3-dicarboxy propyl diphenyl phosphine oxide/decamethylene diamine salt in the copolymerization process, which results in the PA6/PA10,4 copolymer [20]. This approach enables covalent bonding of the FR group, which is potentially highly beneficial for preventing the migration and leaching of flame retardant from the polymer. However, structural changes introduced in the chemical structure of the PA6 back chain produce new challenges regarding the chemical recyclability. The second proposed direction for solving the flammability problem of the PA6 film and textile materials is based on the in situ synthesis of phosphine-oxide-based macromolecules during the PA6 reactive extrusion process [26]. In this case, formation of phosphine-oxide-based oligomeric and low-molecular-weight polymers was found to be beneficial for improved flame retardancy and non-leaching behavior. The authors highlighted that lubricating influence of phosphine oxide macromolecules in PA6 matrix influenced the melt processing and final properties of the fiber, which requires future optimization. The third reported approach includes production of bicomponent PA6 fibers (of 350 μm diameter) with PA6 located in the core and flame-retardant PA6/aluminum diethyl phosphinate/montmorillonite nanoclay composite located in the sheath, or the opposite [27]. The location of flame retardants in the sheath of bicomponent fiber is found to be beneficial for reduced burning behavior. The fourth approach proposed by our research group considers the in situ polymerization of *ε*-caprolactam in the presence of non-reactive 6,6′-(1-Phenylethane-1,2-diyl)bis(dibenzo[c,e][1,2]-oxaphosphinine-6-oxide) (PHED), which is a bridged DOPO derivative [28]. This approach enables one-step production of a linear PA6 back bone with well-dispersed physically incorporated FR in the PA6 matrix, which has proven to be highly beneficial for the effective flame retardancy at lowered FR concentrations. The melt-spinning of the PA6/PHED textile filaments of around 81 μm diameter was conducted without clogging of the filters and spinneret, and with no filament breakage, which enabled production of the partly drawn wounded filaments and knitted fabric as well. However, the incorporated PHED influenced the melt-rheological properties and reduced the tensile properties of the filament, indicating that structural characteristics of the in situ produced PA6/FR system, need to be optimized. 

In order to better understand the influence of the structure of the flame-retardant molecule on the polymerization process and properties of the in situ synthesized PA6, two additional bridged DOPO derivatives were selected according to their potential as FR for PA6. The first one was a P−N bond phosphonamidate derivative, known from the literature as EDA-DOPO, with diamino ethylene bridging two DOPO groups. The second one was from the same P−C bond phosphinate derivative group as PHED, but with a naphthyl group attached to the ethylene bridge connecting the two DOPO molecule structures, instead of benzyl as in the case of PHED. Application of these three FRs in the in situ polymerization process provides an insight into their influence on the morphological, molecular, structural, melt-rheological, and thermal properties of thus produced PA6 polymers, as well as the polymer interactions with the incorporated FRs. These research results are important for the further optimization and development of directions toward future sustainable flame-retardant PA6 textile materials. 

## 2. Experimental 

### 2.1. Materials

*ε*-Caprolactam was kindly supplied by Brüggemann, Belguim. The syntheses of flame retardants were conducted by using 9,10-dihydro-9-oxa-10-phosphaphenanthrene-10-oxide (DOPO, ABCR, 97%, Karlsruhe, Germany), ethylenediamine (Sigma Aldrich, Bratislava, Slovakia, for synthesis), trimethylamine (Sigma Aldrich, 99,5%, Bratislava, Slovakia), sulfuryl chloride (Sigma Aldrich, 97%, Bratislava, Slovakia), potassium hydroxide (Merck, pellets for analysis EMSURE, Bratislava, Slovakia), phosphorus oxychloride (Sigma Aldrich, 99%, ACS Reagent, Bratislava, Slovakia), dichloromethane (Honeywell, ≥99.8%, Sharlotte, NC, USA), 2-acetophenon (Sigma Aldrich, 99% Reagent plus, Bratislava, Slovakia), 2-acetonaphthone (Sigma Aldrich, 99%, Bratislava, Slovakia), xylene (Sigma Aldrich, ACS reagent, Bratislava, Slovakia), dichloromethane (Merck, Bratislava, Slovakia, for analysis EMPARTA^®^ ACS), and isopropyl alcohol (Sigma Aldrich, ≥99.7%, Bratislava, Slovakia). Flame retardants 6,6′-(Ethane-1,2-diylbis(azanediyl))bis(dibenzo[c,e][1,2]oxaphosphinine-6-oxide) (ED), 6,6′-(1-(2-Naphthyl)ethane-1,2-diyl)bis(dibenzo[c,e][1,2]-oxaphosphinine-6-oxide) (NED), and 6,6′-(1-Phenylethane-1,2-diyl)bis(dibenzo[c,e][1,2]-oxaphosphinine-6-oxide) (PHED) were synthesized according to the literature [29,30]. 

**ED**: Yield 80%. ^1^H NMR (DMSO-d6, *δ* (ppm)): 8.10–8.15 (m, 4H), 7.68–7.78 (m, 4H), 7.47–7.54 (m, 2H), 7.36–7.45 (m, 2H), 7.24–7.34 (m, 2H), 7.13–7.19 (m, 2H), 5.72 (dt, 2H), 2.84 (m, 4H). ^31^P NMR (DMSO-d6, *δ* (ppm)): 14.64, 14.67. **NED**: Yield 55%. ^1^H NMR (CDCl_3_, *δ* (ppm)): 6.4–8.0 (m, 23H), 3.7 (m, 1H), and 2.5–3.1 (m, 2H). ^31^P NMR (CDCl_3_) *δ* (ppm)): 30.33, 30.32. **PHED**: Yield: 67%. ^1^H NMR (CDCl_3_, δ (ppm)): 6.7–8.0 (m, 21H), 3.4–3.7 (m, 1H), and 2.5–3.0 (m, 2H). ^31^P NMR (CDCl_3_, δ (ppm)): 34.2–35.9. 

### 2.2. In Situ Polymerization 

**Figure 1:**
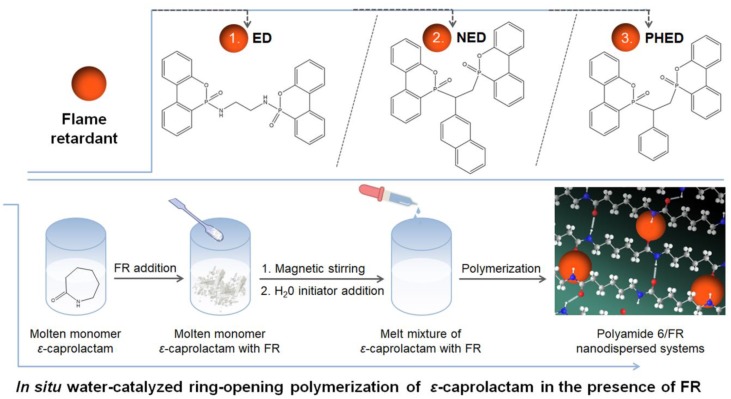
Molecular structures of the (ED), (NED), and (PHED) flame retardants (FRs) and the preparation route for the FR PA6 polyamide 6/FR nanodispersed systems.

PA6/FR systems were prepared by in situ water-catalyzed ring-opening polymerization of the *ε*-caprolactam monomer in the presence of FRs. The polymerization process was performed in a hydrothermal autoclave reactor with a Teflon chamber. First, vacuum-dried *ε*-caprolactam monomer was melted in the Teflon chamber at 180 °C on a temperature-controlled hot plate provided with a magnetic stirrer and placed inside a fume hood under an inert argon atmosphere. Each FR was dispersed in the molten *ε*-caprolactam at a concentration of 10 wt.% under constant stirring. Subsequently, the temperature was gradually reduced to 80 °C, and then 1 wt.% of water was added dropwise. Afterwards, the Teflon chamber was placed in the hydrothermal autoclave reactor, and the mixture was polymerized at 250 °C for 10 h under autogenous pressure. For comparison, the reference PA6 in the absence of FR was synthesized through the same method. The resulting PA6/FR materials containing ED, NED, and PHED were coded as PA6/ED, PA6/NED and PA6/PHED, respectively. Molecular structures of the used FRs and the preparation route for the polyamide 6/FR nanodispersed systems are schematically presented in Figure 1.

### 2.3. Characterization

^1^H and ^31^P NMR spectra were recorded on a Varian 300 MHz NMR spectrometer, model Unity INOVA (Palo Alto, CA, USA). Samples were prepared in deuterated chloroform, CDCl_3_, with tetramethylsilane (TMS) as an internal standard (δ(TMS) = 0.0 ppm). 

^1^H and ^31^P magic-angle spinning (MAS), and ^1^H–^13^C cross-polarization magic-angle spinning (CPMAS) NMR spectra were measured on a 600 MHz Varian NMR system equipped with a 3.2 mm Varian T3 HX MAS probe (Palo Alto, CA, USA). Larmor frequencies for ^1^H, ^31^P and ^13^C nuclei were 599.43 MHz, 242.65 MHz, and 150.74 MHz, respectively. Sample rotation frequency was 20 kHz. In the ^1^H MAS experiments, nuclei were excited by the 90-degree pulse of 2.8 µs, 4 scans were acquired, and recycling delay between consecutive scans ranged from 10 s to 60 s for different samples. In the ^31^P MAS measurements, duration of the 90-degree pulse was 2.2 µs, 8–1000 scans were collected, and recycling delay was 30–240 s. The ^1^H–^13^C CPMAS experiment employed RAMP during the 4 ms CP block and high-power XiX proton decoupling during acquisition; recycling delay was 5–20 s and number of scans ranged from 500 to 1000. Frequency axes of the ^1^H MAS and ^1^H–^13^C CPMAS spectra were referenced to the corresponding signals of TMS, and the ^31^P spectral axis was calibrated relative to the signal of 85% H_3_PO_4_.

Size exclusion chromatography (SEC) was performed in a Knauer SEC with column PL-MiniMix-C using pentafluorophenol/chloroform(PFP/CHCl_3_) (1/2 vol/vol) as eluent with a flow rate of 0.3 mL/min and RI detector.

An X-ray diffractometer (XRD) PANalyticalX’Pert PRO (CuKα1 = 1.5406 Å) with a completely open X’Celerator detector (2.122° 2*θ*) was used for recording the XRD pattern of PA6 (Malvern, UK). The XRD pattern was measured from 5 to 80° 2*θ* with a step size of 100 s.

Scanning electron microscopy (SEM) and energy-dispersive X-ray spectroscopy (EDS) were performed on a Zeiss SUPRA 35VP SEM microscope (Jena, Germany) equipped with an energy dispersive X-ray spectrometer (EDS) Oxford Instruments (Abingdon, UK). The samples were coated with Cr. 

Differential scanning calorimetric (DSC) analysis was used to measure the glass transition (*T*_g_), melting (*T*_m_), and crystallization (*T*_c_) temperatures using a Mettler Toledo (Schwerzenbach, Switzerland) DSC1 instrument at temperatures ranging from 25 to 280 °C (or just above *T*_m_ of each sample) with heating and cooling rates of 10 °C/min in a nitrogen atmosphere at a flow rate of 30 mL/min using aluminum standard 40 µL crucibles with pierced lid. The sample masses varied between 3.2 and 3.9 mg. The second heating run was used for the determination of the *T*_g_ by evaluating a change in the specific heat capacity and using the onset of relaxations at *T*_g_. The obtained *T*_g_ values fit well to the correlation between glass transition and melting temperatures, *T*_g_ ≈ 2/3 ∙ *T*_m_. [31]. The heating runs were analyzed to determine the melting temperatures of the first and second heating runs (*T*_m1_ and *T*_m2_, respectively) of each sample. Furthermore, the first heating run was analyzed to determine degree of crystallization, *X*_c_, using Equation (1):

(1) $$X_{C}=\frac{∆H_{m1}}{∆H_{m}^{{}^{\circ}}\cdot x}$$

where ∆*H*_m1_ is the melting enthalpy from the first heating run, ∆*H^°^*_m_ is the melting enthalpy of a 100% crystalline PA6 reported to be 230 J/g in the fiber production [19], and *x* corresponds to the weight percentage of PA6 polymer in the sample. 

Rheological measurements of the melts were conducted on an Anton Paar Physica MCR 301 rotational rheometer (Graz, Austria), using a parallel-plate geometry with a diameter of 25 mm and a gap of 0.5 mm. The measurement temperature was 225 °C, with a fixed shear strain of 5% and over a frequency range of 0.1 to 500 rad/s.

Thermogravimetric (TG) analyses of 10 mg samples in an air and a nitrogen atmosphere at a gas flow rate of 50 mL/min were performed on a Mettler Toledo (Schwerzenbach, Switzerland) TGA/DSC1 Thermogravimetric Analyzer from 25 to 800° C with a heating rate of 10 °C/min in an open 150 µL platinum pans. Blank curves were subtracted for all measurements. The curves of weight difference between the experimental and theoretical TG curves were calculated according to the method presented by Bourbigot et al. [32]. Thermogravimetric analyses were also used for the determination of *ε*-caprolactam conversion, CL_conv_, in accordance with the method proposed by Zhang et al., 2006 [33].

The UL-94 test was performed in a vertical configuration in the Flammability Cabinet UL 94 by ATS FAAR, Italy. The size of the compression molded bar samples was 130 × 13 × 0.8 mm^3^. 

The limiting oxygen index (LOI) was determined according to EN ISO 4589-2 using the Oxygen Index Apparatus by NOSELAB-ATS (IT). The top surface ignition procedure was conducted on compression molded bar samples of size 130 × 6.5 × 3.2 mm^3^.

## 3. Results and Discussion

In order to investigate the influence of the FR molecular structure on the properties of the prepared PA6/FR systems, three different organophosphorus derivatives were used. The selected bridged DOPO derivatives included: (1) ED, which is a P−N bond phosphonamidate derivative [29], and (2) NED and (3) PHED, which are the P−C bond phosphinate derivatives [28,34]. The DSC heating and cooling runs of the ED, NED, and PHED samples are presented in Figure S1 in Supplementary Materials. During the first heating run, ED melted at 273 °C, and NED and PHED melted at 229 and 188 °C, respectively (Figure S1). Whilst the transparent melt mixture with *ε*-caprolactam was obtained only in the case of the PHED prior the start of polymerization process, it could be suggested that ED and NED were in the melted or rather solubilized form during the PA6 synthesis as polymerization temperature was set at 250 °C and the temperature depression for melting of ED and NED could occur. This is beneficial for the uniform distribution of the applied FRs in the in situ formed PA6 matrix. 

### 3.1. Structure Characterization

**Figure 2:**
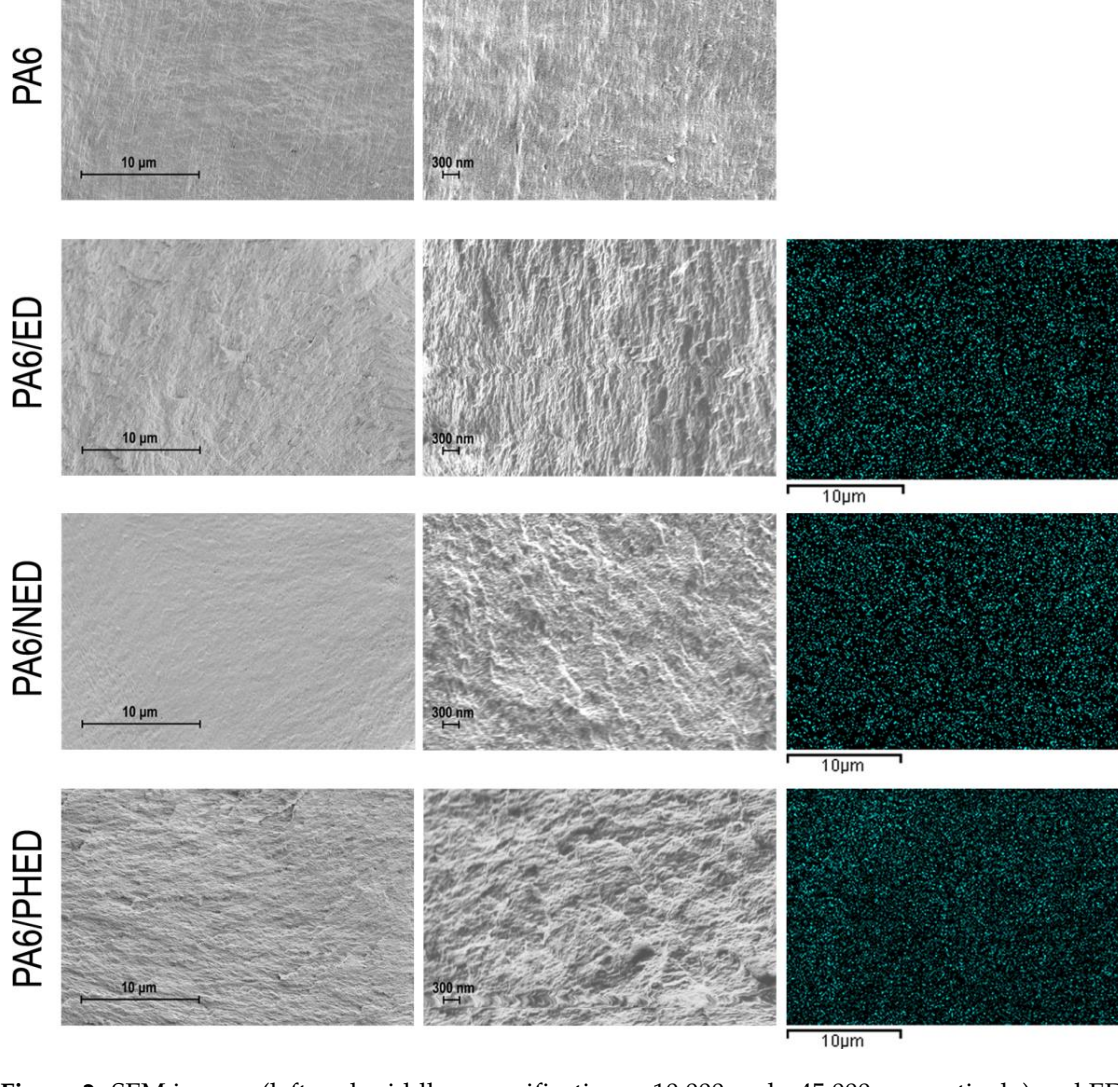
SEM images (left and middle, magnification: ×10,000 and ×45,000, respectively) and EDS phosphorus mapping (right) of the PA6 and PA6/ED, PA6/NED, and PA6/PHED samples.

SEM images and EDS phosphorus distribution maps of the reference PA6 and PA6/ED, PA6/NED, and PA6/PHED samples are shown in Figure 2. SEM images confirmed no visible microagglomerates of the incorporated FRs, and EDS phosphorus mapping confirmed that phosphorus was uniformly distributed in the bulk of all three PA6/ED, PA6/NED, and PA6/PHED samples. These results indicate the production of PA6 with uniformly distributed nanodispersed FRs in the case of all three PA6/ED, PA6/NED, and PA6/PHED samples. The results of the SEM analysis performed at higher magnification showed that incorporated FRs changed the polymer morphological characteristics, indicating their influence on the polymer microstructure. 

Results for the *ε*-caprolactam conversion, CL_conv_, determined from the thermogravimetric analysis, and results for the number-average molecular weight, *M*_n_, the weight-average molecular weight, *M*_w_, and molecular weight distribution, *M*_w_/*M*_n_, obtained from the size exclusion chromatography are shown in Table 1. 

Whilst each FR present in the polymerization system slightly increased conversion of *ε*-caprolactam to PA6 by the comparable extent, the results from the SEC analysis indicate the different extent of their influence on the PA6 molecular weights and their distribution. The presence of ED, NED, and PHED in the polymerization system (1) reduced *M*_n_ for 71.9%, 36.0%, and 19.3%, respectively; (2) reduced *M*_w_ for 48.8%, 20.2%, and 7.7%, respectively; and (3) increased *M*_w_/*M*_n_ for 82.1%, 24.7%, and 14.5%, respectively. According to these results, it can be concluded that among the applied FRs, ED significantly hindered the polymerization process, whilst PHED compound provided minimal influence. In the case of ED, the reason for the significant reduction of PA6 molecular masses and broadening of their distribution could be ascribed to hydrogen bonding between the NH from the ED phosphonamidate group and carbonyl group from *ε*-caprolactam, which could hinder the chain propagation in addition reaction as well as to participation of ED in the polymerization reaction as chain terminator specie. Despite NED and PHED compounds not possessing reactive chemical groups in their structures, their bulky molecules could also hinder the condensation between polyamide chains. As PHED and NED have benzyl and naphthyl groups, respectively, attached to the ethylene bridge connecting two DOPO groups, it can be suggested that less steric hindrance is provided by the PHED additive. 

**Figure 3:**
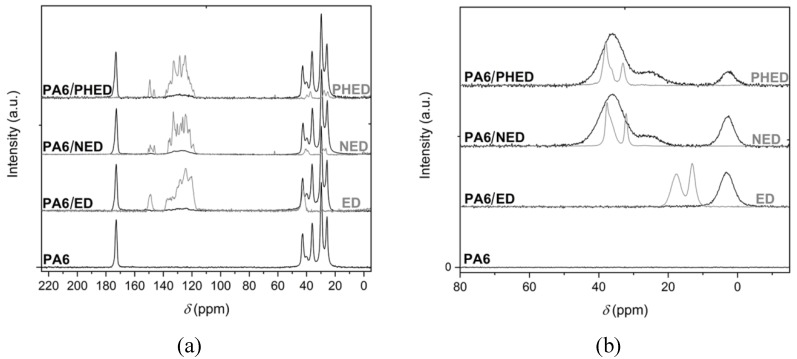
(**a**) ^1^H–^13^C CP/MAS and (**b**) ^31^P MAS NMR spectra of PA6 and PA6/ED, PA6/NED, and PA6/PHED samples.

The molecular structures of the PA6 corresponding to the reference PA6, PA6/ED, PA6/NED, and PA6/PHED samples were investigated by solid state ^1^H–^13^C CP/MAS NMR. Structures of the FRs before and after incorporation in the PA6 matrix were investigated by solid state ^31^P MAS NMR spectroscopy. The obtained spectra are presented in Figure 3. The signals in the ^1^H–^13^C CP/MAS NMR spectra (Figure 3a) corresponding to the PA6 sample, as well as to the PA6/ED, PA6/NED, and PA6/PHED samples, can be assigned to the typical signals of the PA6 in α-crystalline form [35,36]. No significant differences could be observed in the solid state ^1^H–^13^C CP/MAS NMR spectra corresponding to the reference PA6, PA6/ED, PA6/NED, and PA6/PHED samples. The ^31^P MAS NMR spectrum of the ED sample (Figure 3b) shows two signals at *δ* = 17.55 and 13.09 ppm, for the P atoms in different surroundings belonging to two diastereomers. In the case of the NED and PHED samples, the ^31^P MAS NMR signals appeared at higher frequencies, where signals at 37.64 and 32.14 ppm for NED, and at 38.0 and 33.04 for PHED, were attributed to the phosphorus atom in the phosphinate group. Comparison of the ^31^P MAS NMR signals in the spectra of the ED, NED, and PHED, with those of the PA6/ED, PA6/NED, and PA6/PHED, shows broadening of signals indicating interactions of additives with PA6 matrix (relaxation of P atoms caused by hydrogen bond formation).

Interactions of additives with PA6 chains also caused shifting of the ^31^P MAS NMR signals to somewhat lower frequencies, which can be observed as the appearance of signals at 36.0 and 25.7 ppm for NED and signals at 36.0 and 24.6 ppm for PHED (Figure 3b). Additionally, in the case of all additives, a new signal at 2.9 ppm appeared, and it was the most prominent in the case of the ED additive. The new P signal indicates the occurrence of strong interaction, or even chemical reaction on the ED’ phosphorus atom during polymerization of *ε*-caprolactam. 

**Figure 4:**
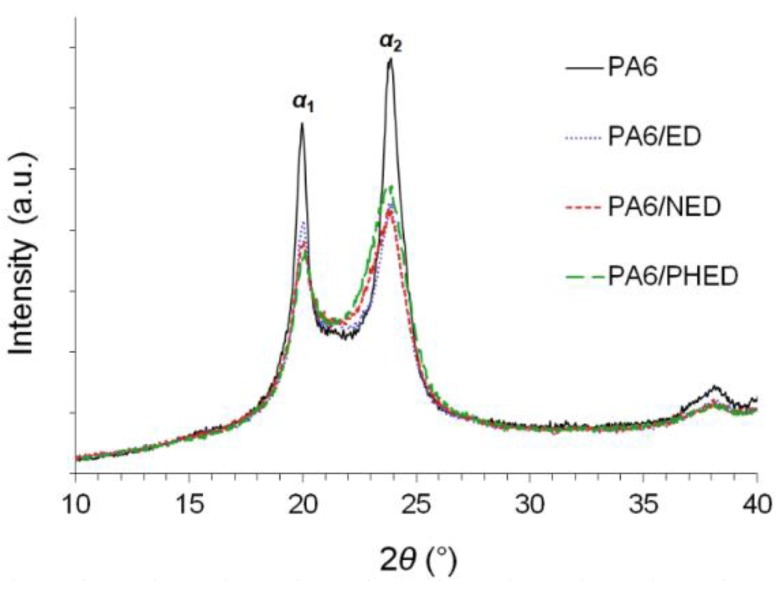
XRD patterns in the range of 10°–40° for PA6 and PA6/ED, PA6/NED, and PA6/PHED samples.

The obtained XRD patterns (Figure 4) for the PA6, as well as for the PA6/ED, PA6/NED, and PA6/PHED showed two diffraction peak maxima located at 2*θ* = 20.0° and 2*θ* = 23.9°, which can be assigned to *α*_1_ and *α*_2_ crystalline phases, respectively. This confirmed successful retention of the *α* crystal form for the PA6 synthesized in the presence of the FRs. The presence of the FRs in the polymerization system decreased degree of crystallinity, which was further quantitatively evaluated from the results obtained in the DSC measurements. 

**Figure 5:**
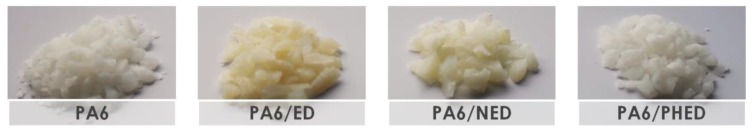
Photographs of the PA6, PA6/ED, PA6/NED and PA6/PHED samples.

The photographs of the PA6, PA6/ED, PA6/NED, and PA6/PHED samples are presented in Figure 5. Although all three FRs were applied in the white powder form, the white color was preserved only in the case of the PA6/PHED sample, whereas the PA6/ED and PA6/NED samples displayed visible yellowing. The yellowing can be caused by different processes, but most of these involve different kinds of reactions towards polymer end groups, e.g., the oxidation process of amine end groups in the presence of water at high-temperature conditions or chemical reaction on the ED’s and NED’s phosphorus atoms during polymerization. Since PA6/ED has the lowest polymer molecular weight among presented materials, it would be expected that it also has the highest amine end group content, and therefore the highest observable yellowing effect. Additionally, the presence of phosphonamidate groups might have an amplifying effect on the yellowing of the PA6 matrix material. 

### 3.2. Melting and Crystallization Behavior

**Figure 6:**
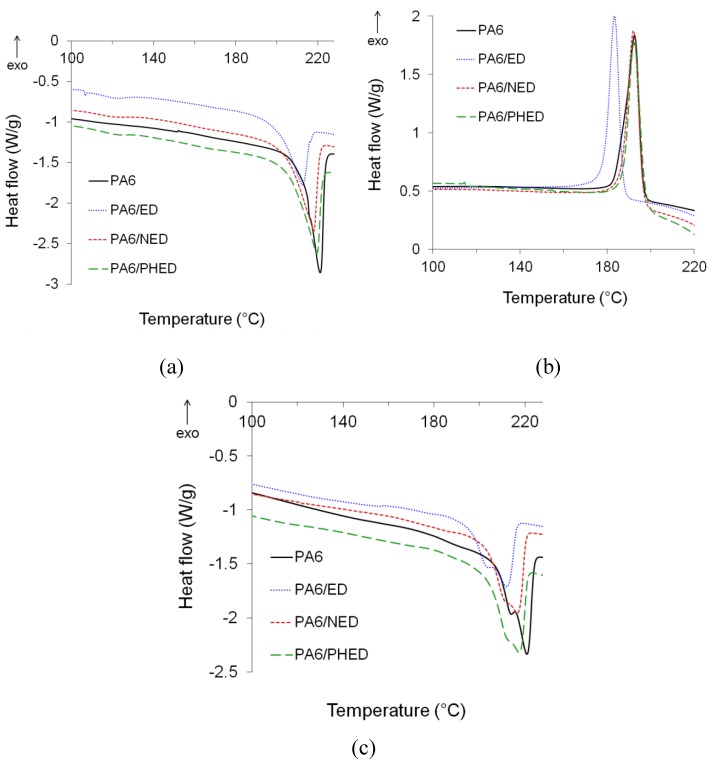
(**a**) Melting temperature (*T*_m1_) from the first DSC heating run; (**b**) crystallization temperature (*T*_c_) from the first DSC cooling run; (**c**) melting temperature (*T*_m2_) from the second DSC heating run for PA6 and PA6/ED, PA6/NED, and PA6/PHED samples.

The DSC curves are presented in Figure 6 and the results for characteristic glass transition, *T*_g_, melting, *T*_m_, and crystallization, *T*_c_, temperatures, as well as for the degree of crystallinities, are summarized in Table 2. The endothermic melting peaks from the first and the second heating runs for the PA6 reference appeared at 222 and 220 °C, respectively, whilst a shoulder at 214 °C appeared on the melting peak from the second heating run (Figure 6a,c). This shoulder was caused by the non-isothermal recrystallization in the DSC measurements [37] and may be attributed to the *γ*-crystalline phase, as well as to *α*-crystallites of different size and perfection [38,39]. A similar phenomenon can also be observed for the PA6/ED, PA6/NED, and PA6/PHED samples. 

The single melting peaks from the first heating runs (Figure 6a) confirmed the *α*-crystalline forms in the reference PA6, as well as in the PA6/ED, PA6/NED, and PA6/PHED, which is in correlation with the results obtained by the solid state ^1^H–^13^C CP/MAS NMR and XRD measurements.

Whilst NED and PHED did not markedly influenced *T*_m1_ and *T*_m2,_ ED caused a decrease of both *T*_m1_ and *T*_m2_ in comparison to the reference PA6, suggesting hindered hydrogen bonding between PA6 chains. Furthermore, PHED and NED did not markedly influenced temperature of PA6 crystallization, whilst in the case of the PA6/ED, the *T*_c_ decreased for 12 °C. The start of crystallization at a lower temperature for the PA6/ED was also accompanied by a lowered degree of crystallinity, *X*_c_, by approximately 35% as well as by the increased *T*_g_ by 7 °C compared to a reference PA6. Contrary to ED flame retardant, NED and PHED did not markedly change *T*_g_, and caused lowering of the *X*_c_ by approximately 2.6% and 15%, respectively, indicating lesser influence on the degree of crystallinity and almost no influencing of the amorphous phase characteristics. These results confirmed that NED and PHED minimally influenced the hydrogen bonding between chains, melting behavior, crystalline structure, and amorphous phase of the in situ formed PA6, which is in correlation with the PA6 molecular masses and their distributions obtained by the SEC analysis. In the case of the PA6/ED sample, the detected changes of the PA6 structural characteristics could also be assigned to the significant changes of the PA6 molecular structure introduced by the ED’s molecules during the polymerization process.

### 3.3. Melt-Rheology 

**Figure 7:**
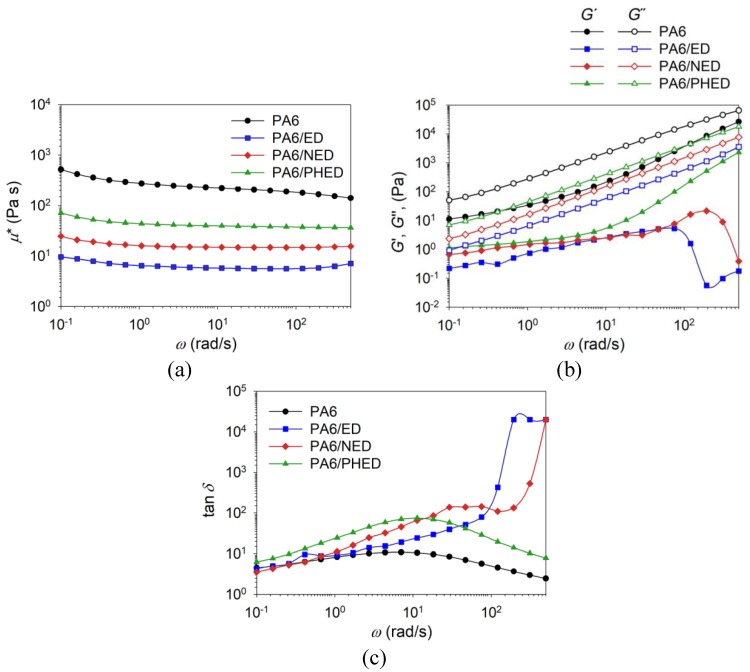
(**a**) Complex viscosity (μ*); (**b**) storage (*G*’) and loss (*G*’’) modulus, and (**c**) loss factor (tan *δ*) plotted logarithmically as a function of the angular frequency (*ω*) for PA6 and PA6/ED, PA6/NED, and PA6/PHED samples.

Melt-rheology measurements were conducted in order to evaluate the impact of the in situ incorporated FRs on the visco-elastic behavior of the PA6 melt. The results for complex viscosity, μ*, storage modulus, *G*’, loss modulus, *G*’’, and loss factor, tan *δ,* are shown in Figure 7. The complex viscosity of the reference PA6 decreased with the increase of angular frequency, *ω*, showing the shear thinning behavior (Figure 7a). It can be seen that incorporated FRs induced a decrease of the complex viscosity of the PA6/ED, PA6/NED, and PA6/PHED samples also showing lower sensitivity to the applied shear strain at angular frequencies higher than 1 rad/s. Decreased complex viscosity of the PA6/ED, PA6/NED, and PA6/PHED melts indicates lubrication effect due to reduced internal friction of molecular chain segments caused by shortening of the PA6 chains, as well as by sterically hindered attraction between the PA6 chains.

The least pronounced decrease of the complex viscosity compared to that of the reference PA6 was detected in the case of the PA6/PHED and the most pronounced was detected in the case of the PA6/ED. In the case of all studied samples, the responses of the elastic, *G’*, and viscous, *G’’*, portions to the applied angular frequencies show no intersection of the moduli, with *G’’* values higher than *G’*, which confirms the viscoelastic characteristics (Figure 7b). Incorporated additives reduced both elastic and viscous components of the PA6 melt, whilst among the applied FRs, PHED provided minimal decrease and did not defect the gradually increased tendency of the PA6 storage modulus over the applied angular frequency range. In the case of PA6/NED and PA6/ED samples, the microstructure broke above 10^2^ rad/s, which is indicated by sudden decline in *G’* modulus. The values of the loss factor (tan *δ*) increased due to the incorporated FRs, because of the higher ratio of the viscous to the elastic portion of the viscoelastic deformation, i.e., intensified domination of the viscous behavior over the elastic behavior (Figure 7c). The incorporated FRs and their influence on the PA6 chain length and the attractive interactions between the PA6 chains contributed to the higher ratio of the viscous to the elastic portion and caused deviation of the rheological behavior from that of the reference PA6. Whereas the reasonable decrease of the complex viscosity can be considered as beneficial for the processability, we have already shown in our previous work [28] that decreased viscosity in the case of the PA6/PHED in comparison to that of the reference PA6 does not markedly hinder the good melt-spinning processing characteristics, but rather influences the tensile properties. In the case of PA6/ED and PA6/NED samples, melt-spinning was not feasible because of the significant reduction of PA6 molecular masses.

### 3.4. Thermal Stability

**Figure 8:**
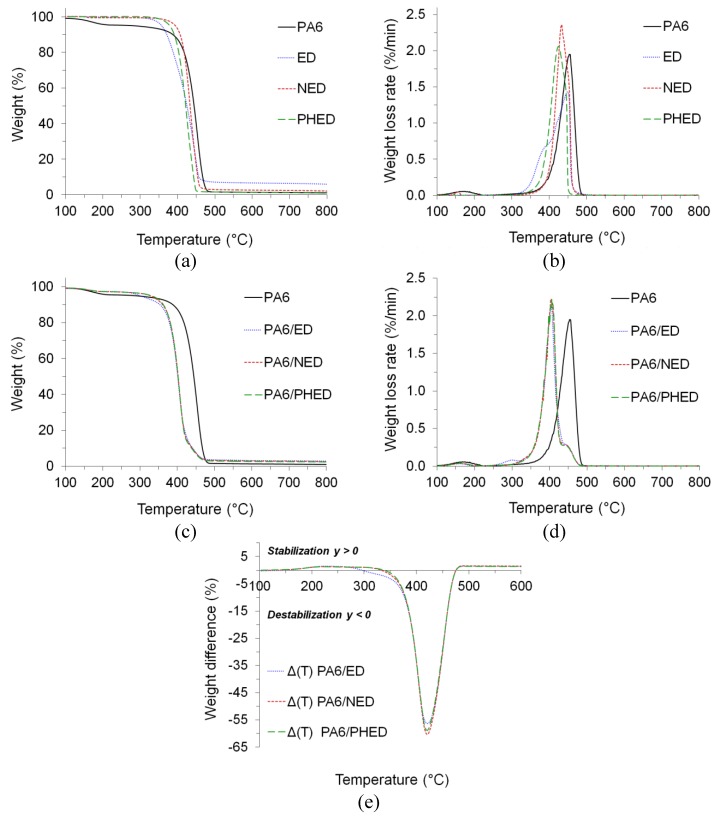
(**a**) TG graphs and (**b**) dTG graphs of PA6 and ED, NED, and PHED samples, (**c**) TG graphs and (**d**) dTG graphs of PA6 and PA6/ED, PA6/NED, and PA6/PHED samples analyzed in nitrogen atmosphere, and (**e**) the weight difference between the experimental and calculated TG curves for PA6/ED, PA6/NED, and PA6/PHED samples.

It has already been shown that ED, NED, and PHED act as flame retardants mainly in the gas phase [28,34,40], which is beneficial for increasing the PA6 flame retardancy. The efficiency of the latter is closely related to the temperature at which flame-retardant volatile radical scavengers are produced, i.e., the premature as well as delayed decomposition of the gas phase flame retardant in comparison to that of the polymer provides inefficient inhibition of the flaming combustion process [8]. In order to clarify if their decomposition temperatures match the PA6 pyrolysis specifics, heat-induced thermal decompositions of PA6 and flame retardants ED, NED, and PHED were analyzed (Figure 8 and Table S1). In comparison to the initial decomposition temperature, *T*_onset_, of the PA6 in nitrogen environment, the *T*_onset_ of the ED, NED, and PHED was 53 °C lower, 6 °C higher, and 20 °C lower, respectively (Figure 8a,b). Temperature of the maximum in the weight loss rates, *T*_max_, for the ED, NED, and PHED were 4, 20, and 30 °C lower, respectively, in comparison with that of the PA6. Higher weight loss rates of the NED and PHED in comparison to the ED demonstrates more accelerated decomposition, which may indicate more intensive production of the flame-retardant volatile radical scavengers. Thermal decomposition of ED’s diastereoisomers occurred via two decomposition steps in the TG curve and a maximum with a shoulder at approximately 395 °C in the first derivative curve. This shoulder (Figure 8b) appeared at a temperature almost 60 °C lower in comparison to *T*_max_ of the PA6, which consequently caused more than two times lower ED residue at *T*_max_ in comparison with those of the NED and PHED (Table S1). Furthermore, higher ED residue at 600 °C in comparison with those of the NED and PHED indicates its higher thermal stability. 

In spite of the differences in the decomposition temperatures of the applied ED, NED, and PHED, the incorporated FRs produced comparable effects on the PA6 thermal stability (Figure 8c,d). Whilst *T*_onset_ values for PA6/ED, PA6/NED, and PA6/PHED were approximately comparable to each other, as were *T*_max_ values, both the *T*_onset_ and *T*_max_ for all three FRs were shifted to temperatures approximately 40 and 50 °C, respectively, lower compared to the *T*_onset_ and *T*_max_ of the PA6. This indicates that interactions between decomposing FRs and the PA6 matrix promote the heat induced pyrolysis of the PA6. According to the results of the weight difference between the experimental and calculated TG curves (Δ*T*, presented in Figure 8e), the obtained negative curves indicate destabilization of the PA6 during the whole anaerobic pyrolysis [32,41]. In the case of the PA6/ED sample, the first derivative curve revealed an additional peak at approximately 302 °C, which preceded the temperature of the maximum in the weight loss rate. In the case of all three PA6/ED, PA6/NED and PA6/PHED, the *T*_max_ was followed by the additional decomposition step at *T*_max,add_ (Figure 8d and Table S1). The latter are close to *T*_max_ values of the ED, NED, and PHED, and the residues at *T*_max,add_ are approximately equal to 10 wt.%, which could be the concentration of the applied FRs. However, our previous results of the TG-FTIR coupled analysis of the PA6/PHED system confirmed releasing of the active phosphorus species during both *T*_max_ and *T*_max,add_. Therefore, interactions between decomposition products of FRs and PA6 led to the formation of the primary char, which underwent further decomposition during the second pyrolysis step. As all three FRs have a dominant gas-phase mode of action, the residues at the end of the forced heat induced pyrolysis process are rather low. However, the residues of the PA6/ED, PA6/NED, and PA6/PHED were two times higher than that of the PA6. Based on these results, it can be assumed that all three used FRs will promote the PA6 decomposition during the forced flaming combustion, but during the limited contact time with the flame, the released gas-phase radical scavengers from decomposing FRs could effectively quench the combustible radicals.

**Figure 9:**
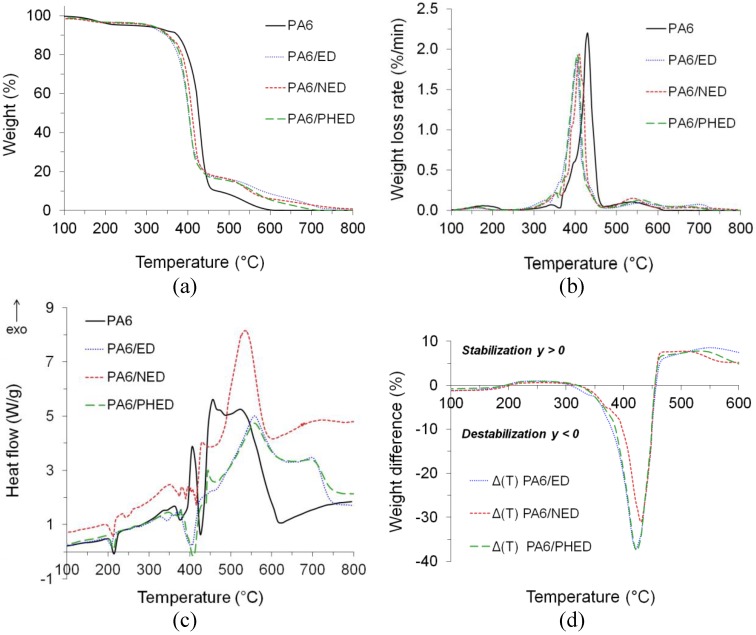
(**a**) TG, (**b**) dTG, and (**c**) DSC graphs of PA6 and PA6/ED, PA6/NED, and PA6/PHED samples analyzed in air atmosphere, and (**d**) the weight difference between the experimental and calculated TG curves for PA6/ED, PA6/NED, and PA6/PHED samples.

In the stage before flaming combustion occurs, the material is exposed to heat and chemically reacts with oxygen. Additionally, after flameout, the residue undergoes a thermo-oxidative decomposition [42]. When heat-induced decomposition is supported by oxygen, decomposition products chemically react with oxygen, which consequently shifted the initial decomposition temperatures to lower values for PA6, and PA6/ED, PA6/NED and PA6/PHED (Figure 9a,b, and Table S2) in comparison to those in the anaerobic pyrolysis. Similarly, as during the pyrolysis, the incorporated ED, NED, and PHED accelerated the start of the decomposition. Furthermore, the air environment caused shifting of the PA6’s *T*_max_ to a lower value, which corresponds to the *T*_max1_. However, the *T*_max1_ values for the PA6/ED, PA6/NED, and PA6/PHED remained almost the same as the *T*_max_ values during the anaerobic pyrolysis. This may indicate that phosphorus radical scavengers emerging from decomposing FRs retarded the gas-phase oxidation reactions. This finding is also supported by the reduced weight loss rates for PA6/ED, PA6/NED, and PA6/PHED during the first decomposition step in comparison to that of the PA6, as well as by the results of the DSC analysis conducted simultaneously with thermo-oxidative decomposition (Figure 9c). 

The latter indicates the endothermic heat effects during the first decomposition step in the case of the PA6/ED and PA6/PHED. Oxygen-assisted decomposition at higher temperatures of the char formed during the first decomposition step further led to the oxidation of residue to volatiles. Whilst this step for PA6 ended at about 600 °C with almost no residue, the incorporated FRs increased thermo-oxidative stability of the char, which resulted in extended char oxidation up to approximately 720 °C and increased char residue up to 8.6 wt.% at 600 °C. The results of the weight difference between the experimental and calculated TG curves (Δ*T*, presented in Figure 9d) confirmed stabilization of the PA6/ED, PA6/NED, and PA6/PHED systems above 450 °C, which resulted in the approximately 50% higher residues at 500 °C in comparison to that of the PA6.

### 3.5. Vertical Flammability Tests

Despite the fact that the applied FRs provided a relatively comparable effect on the PA6 thermal and thermo-oxidative stability, among the applied FRs, PHED minimally interfered with the PA6 polymerization process and minimally affected the PA6 molecular and crystalline structure and melt-rheology. Accordingly, further testing of the downward flame spreading (limited oxygen index, LOI) and vertical upward burning (UL94) was conducted on the PA6/PHED. To this purpose, the tested samples included the PA6 sample with PHED incorporated at a concentration of 15 wt.% (PA6/15PHED) in addition to the sample with PHED incorporated at concentration of 10 wt.% (PA6/10PHED). The obtained results are summarized in Table 3. The LOI values for the 3.2 mm tick bar PA6, PA6/10PHED, and PA6/15PHED samples were equal to 25.0%, 26.9%, and 27.8%, respectively. The incorporated PHED increased LOI, which increased with the PHED’s concentration. The results of the UL94 vertical flammability testing of 0.8 mm tick bar samples showed that the flame-retardant action of the decomposing PHED substantially reduced flammability of the dripping and prevented ignition of the cotton indicator. In comparison to reference PA6, the flaming combustion of the PA6/PHED samples during the application of the burner flamer showed stronger gleaming and was accompanied by the melt bubbling before dripping. This indicates transport of PHED’s decomposing volatiles from the condensed to the gas phase, where phosphorus radical scavengers retarded the combustion process, which resulted in non-flammable dripping. These results are in good correlation with the results obtained for the thermal decomposition processes in nitrogen and air environments, confirming that although the incorporated PHED accelerated PA6 decomposition, its flame-retardant action increased the stability of the char residue during the oxygen-assisted thermal decomposition. 

## 4. Conclusions

In this research, we investigated the perspective of a one-step process of PA6 polymerization with simultaneous incorporation of FRs. Three different bridged DOPO derivatives, i.e., a P−N bond phosphonamidate derivative, ED, and two P−C bond phosphinate derivatives, NED and PHED, were used in the in situ PA6 polymerization process. The results showed that this approach enabled production of the PA6 with uniformly distributed nanodispersed flame retardants in the case of all three applied ED, NED, and PHED flame retardants. Although the pyrolysis specifics of the used FRs differ to each other to a certain extent, all three ED, NED, and PHED provided comparable effects on PA6’s thermal stability. Despite that these gas-phase active FRs shifted PA6 decomposition to lower temperatures, the released gas-phase radical scavengers from decomposing FRs effectively retarded the gas-phase oxidation reactions, which increased thermo-oxidative stability of the char formed during the first decomposition step and resulted in the approximately 50% higher residues at 500 °C in comparison to that of the raw PA6. Evaluation of the molecular and structural characteristics of the PA6 synthesized in the presence of the FRs, against that of the raw PA6, highlighted the strong dependence of the final PA6 properties on the FR molecular structure. The PA6 molecular and structural characteristics were maximally affected by ED and minimally affected by PHED. The strong inhibition of the PA6 polymerization in the presence of ED could be ascribed mainly to the hindered chain propagation due to the hydrogen bonding between the NH from the phosphonamidate group and carbonyl group from *ε*-caprolactam. In the case of NED and PHED compounds, which do not possess reactive chemical groups in their structures, naphthyl and benzyl groups, respectively, attached to the ethylene bridge connecting two DOPO groups, produced steric effects that hindered condensation between polyamide chains. This effect was less pronounced in the case of the less bulky PHED molecule in comparison with NED. Accordingly, PHED provided the smallest effect on the reduction of the complex viscosity, and consequently, on the PA6 a melt-spinning characteristic, whilst melt-spinning in the case of the ED and NED was not feasible. The nanodispersed state of the PHED in the PA6 matrix provided an effective flame-retardant effect, which substantially reduced flammability of the dripping and prevented ignition of the cotton indicator in the UL94 testing of 0.8 mm tick bar samples with PHED concentration equal to 15 wt.%. These results highlight that the in situ polymerization can provide a successful solution for the agglomeration problem of the FRs in the PA6 matrix and also enables lowering the concentration of the applied FR, whilst pertaining the flame-retardant efficiency at the highest level. To support the gas phase activity of PHED with protection in the condensed phase as well as to introduce a reinforcing effect to improve fiber tensile properties, the currently ongoing research includes application of the PHED additive with simultaneous incorporation of anti-dripping char forming, smoke suppressing and reinforcing additives. 

## Figures and Tables

**Table 1:** *ε*-Caprolactam conversion, and molecular weight of PA6 and PA6/FR samples.

Sample	CL_conv_ (%)	*M*_n_ (g/mol)	*M*_w_ (g/mol)	*M*_w_/*M*_n_
PA6	95.9	7.1 × 10^4^	2.6 × 10^5^	3.57
PA6/ED	97.4	2.0 × 10^4^	1.3 × 10^5^	6.50
PA6/NED	97.3	4.6 × 10^4^	2.0 × 10^5^	4.45
PA6/PHED	97.2	5.8 × 10^4^	2.4 × 10^5^	4.09

**Table 2:** Glass transition, melting and crystallization temperatures, and degrees of crystallinity.

Sample	*T*_g_ (C°)	*T*_m1_ (C°)	*T*_m2_ (C°)	*T*_c_ (C°)	*X*_c_ (%)
PA6	52	222	220	196	31.2
PA6/ED	59	213	212	184	20.4
PA6/NED	53	220	217	194	30.4
PA6/PHED	52	221	219	194	26.6

**Table 3:** The results of the limiting oxygen index (LOI) and UL94 tests performed on 0.8mm tick, respectively, PA6, PA6/10PHED, and PA6/15PHED bar samples.

Sample	P (%)	LOI (%)	UL 94				
			Test	*t*_1_/*t*_2_ (s)	Cotton Ignition	Burning up to the Clamp	Classification
PA6	0	25.0	1	0/0	Yes	No	V2
			2	0/0	Yes	No	V2
PA6/10PHED	1.16	26.9	1	1/1	No	No	V0
			2	1/0	Yes	No	V2
PA6/15PHED	1.74	27.8	1	1/0	No	No	V0
			2	0/1	No	No	V0

## Supplementary Materials

The following are available online at https://www.mdpi.com/2073-4360/12/3/657/s1, Figure S1: DSC runs of the ED (a), NED (b) and PHED (c) samples., Table S1: TG data for PA6 and ED, NED, PHED and PA6/ED, PA6/NED, and PA6/PHED samples obtained under nitrogen atmosphere., Table S2: TG data for PA6 and PA6/ED, PA6/NED, and PA6/PHED samples obtained under air atmosphere.
