# Supplementary material for: Effect of Different Flame-Retardant Bridged DOPO Derivatives on Properties of in Situ Produced Fiber-Forming Polyamide 6
Source: Polymers (Basel). 2020 Mar 13;12(3):657. doi: 10.3390/polym12030657 (PMC7183073; doi:10.3390/polym12030657)
Supplement: Supplementary file 1 [file polymers-12-00657-s001.pdf]

Supporting Information

Effect of Different Flame-Retardant Bridged DOPO Derivatives on Properties of in Situ Produced Fiber-Forming Polyamide 6

Jelena Vasiljević [1,*] Marija Čolović [2,*], Nataša Čelan Korošin [3], Matic Šobak [2], Žiga Štirn [2] and Ivan Jerman [2]

1 Faculty of Natural Sciences and Engineering, University of Ljubljana, Aškerčeva 12, 1000 Ljubljana, Slovenia
2 National Institute of Chemistry, Hajdrihova 19, 1000 Ljubljana, Slovenia; Matic.Sobak@ki.si (M.Š.); Ziga.Stirn@ki.si (Ž.Š.); ivan.jerman@ki.si (I.J.)
3 Faculty of Chemistry and Chemical Technology, University of Ljubljana, Večna pot 113, 1000 Ljubljana, Slovenia; natasa.celan@fkkt.uni-lj.si
* Correspondence: jelena.vasiljevic@ntf.uni-lj.si (J.V.); Tel: +386 1 20 03 200 (J.V.); Fax: +386 1 20 03 270 (J.V.); marija.colovic@ki.si (M.Č.); Tel: +386 1 476 0 323 (M.Č.)

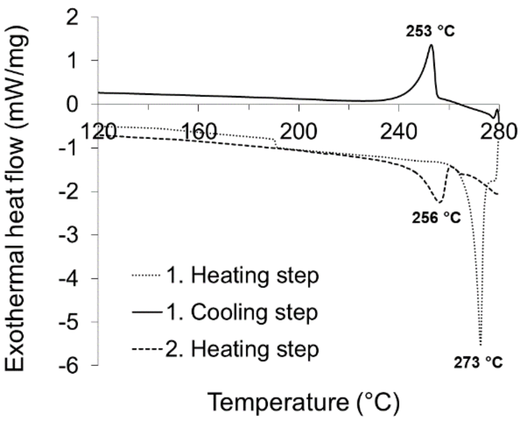

(a)

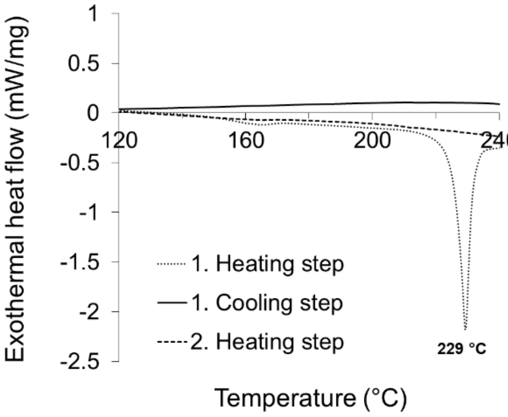

(b)

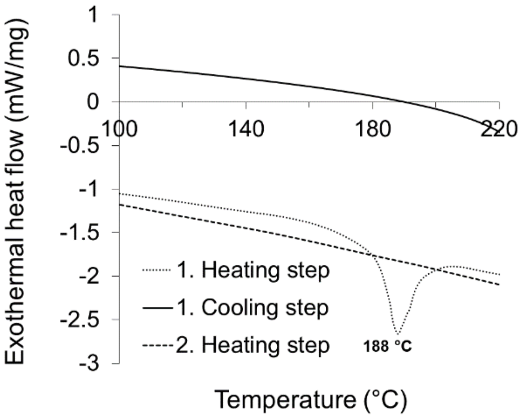

(c)

Figure S1. DSC runs of the ED (a), NED (b) and PHED (c) samples.

Table S1. TG data for PA6 and ED, NED, PHED and PA6/ED, PA6/NED, and PA6/PHED samples obtained under nitrogen atmosphere.

Sample	T_{onset} (℃)	T_{max} (℃)	Residue at T_{max} (%)	$T_{max,add}$ (℃)	Residue at $T_{max,add}$ (%)	Residue at 600 ℃ (%)
PA6	418	453	33.9	-	-	1.3
ED	365	449	19.8	-	-	6.6
NED	412	433	50.9	-	-	2.6
PHED	398	424	45.2	-	-	1.3
PA6/ED	377	405	41.6	443	9.5	3.2
PA6/NED	378	404	45.9	439	10.4	2.9
PA6/PHED	377	406	41.1	437	10.4	2.6

Table S2. TG data for PA6 and PA6/ED, PA6/NED, and PA6/PHED samples obtained under air atmosphere.

Sample	T_{onset} (℃)	T_{max1} (℃)	Residue at T_{max1} (%)	T_{max2} (℃)	Residue at T_{max2} (%)	Residue at 600 ℃ (%)
PA6	318	430	41.5	535	5.1	0.3
PA6/ED	293	405	44.0	560	11.9	8.6
PA6/NED	317	410	50.1	535	12.3	6.1
PA6/PHED	325	405	45.7	555	10.4	5.5
